# The Odontocete Ear Canal-Associated Lymphoid Tissue (ECALT) and Lymph Nodes: Morphological and Pathological Description with Immuno-Phenotypic Characterisation

**DOI:** 10.3390/ani12172235

**Published:** 2022-08-30

**Authors:** Steffen De Vreese, Cinzia Centelleghe, Jean-Marie Graïc, Giorgia Corrazola, Lonneke L. IJsseldijk, Michel André, Sandro Mazzariol

**Affiliations:** 1 Department of Comparative Biomedicine and Food Science, University of Padova, 35020 Legnaro, Italy; 2 Laboratory of Applied Bioacoustics, Technical University of Catalunya (BarcelonaTech), 08800 Vilanova i la Geltrú, Spain; 3 Division of Pathology, Department of Biomolecular Health Sciences, Faculty of Veterinary Medicine, Utrecht University, 3584CL Utrecht, The Netherlands

**Keywords:** lymphoid system, external ear canal, odontocetes, immunohistochemistry, MALT, lymph node

## Abstract

**Simple Summary:**

The marine mammal immune system is of vital importance for the health of any marine mammal. With changes in the natural environment and with increasing anthropogenic stressors such as pollution, the immune system is challenged to unknown extents. Dolphins and other odontocete cetaceans have been shown to be particularly sensitive to anthropogenic influence in many aspects. In this regard, it is important to understand how these animals cope with novel stressors and how the immune system works and responds. In studying parallel issues related to underwater noise pollution, we looked at the cetacean ear canal and analysed in detail the cells of the immune system. Like the skin, it is likely to be exposed to the external environment and requires a local defence system as a first barrier to incoming threats. We studied the ear-canal associated immune system and describe the cell population using a variety of microscopic techniques. We describe healthy and activated tissue and cases with inflammation of the external ear canal and compare the different physiological states. As such, this study contributes to acquiring a general understanding of the odontocete cetacean immune system.

**Abstract:**

A changing marine environment with emerging natural and anthropogenic stressors challenges the marine mammal immune system. The skin and adnexa form a first protective barrier in the immune response, although this is still relatively understudied in cetaceans. The cellular and tissue morphology of the nodular and diffuse lymphoid tissue are not fully charted and the physiological responses are not yet completely understood. The odontocete’s external ear canal has a complex relationship with the external environment, with an artificial lumen rendering the inside of the canal a relatively secluded environment. In this work, we studied the odontocete ear canal-associated lymphoid tissue (ECALT) by histo- and immunohistochemistry (HC, IHC) with anti-CD3, anti-CD20, anti-Iba-1, anti-HLA-DR, and anti-vimentin antibodies. The ECALT cellular composition consists mainly of B-lymphocytes with the occasional presence of T-lymphocytes and the dispersed distribution of the macrophages. In cases of activation, the cellular reaction showed a similar pattern with the occasional presence of T-cells, plasma cells, and neutrophils. Nodular lymphoid tissue was generally in line with the description in other odontocetes, although with abundant erythrocytes throughout the entire organ. This study contributes to the understanding of the cellular composition of diffuse and nodular lymphoid tissue in several species of odontocetes, and in association with inflammation of the external ear canal.

## 1. Introduction

For the past several decades, there has been an increasing concern regarding the severity of the negative impact of anthropogenic contaminants on the immune system responses and the associated health status of marine mammals [1,2]. Investigation of the immunological and immunopathological mechanisms in cetaceans is of importance to understand the immune response to infectious diseases such as cetacean morbillivirus [3,4,5]. In this regard, an understanding of the morphological characteristics and cellular composition of lymphoid organs is essential to gaining insights into cetacean immunology.

There are many homologies between the terrestrial and marine mammal immune system [1]. As in all mammals, cetaceans possess lymphoid organs that are spatially distributed in strategic locations as part of a first barrier protection mechanism against infection [1,6,7]. Aside from nodular lymphoid tissue, several organs possess a mucosa-associated lymphoid tissue (MALT), which is diffusely spread lymphoid tissue along mucosal surfaces, essential in initiating an immune response to extraneous antigens [8]. In several species of small odontocetes, MALT sites have been determined including the organized tonsils in rrprthe larynx, spread clusters of lymphoid cells in the skin, uterine cervix, gastrointestinal tract, and the anal tonsil [7,9,10,11]. Although the odontocete MALT was concluded to be similar to the one in terrestrial mammals, the cellular composition is yet to be fully understood [11]. For this purpose, immunohistochemical techniques for the cellular identification of lymphoid and antigen-presenting cells and macrophages have been successfully applied to a large variety of odontocetes, while there is still a large gap in knowledge on the immunological characteristics of the cetacean skin and annexes [4,11,12,13,14,15].

In odontocetes, there is a connection between the ear canal lumen and exterior [16]. Although recent histological research demonstrates a complex morphological conformation that allows for the flow of the ear canal luminal content (cerumen and cellular debris) into the outside world, there is a lack of knowledge on the physiological processes involved. In histological slides, the ear opening can appear obstructed with epithelial and glandular content, continuous but with a minute lumen, or artificially closed with ‘collapsed’ epithelial walls, rendering the ear canal sensitive to exposure to the external environment with contaminants and infectious agents [16].

This study focused on contributing to the knowledge of the immune system in the cetaceans, specifically with a novel description of a distinct ear-canal associated lymphoid tissue and lymph nodes including the identification of the cellular composition with immunohistochemical markers, and the description and link with ear canal pathologies in various wild odontocetes.

## 2. Materials and Methods

### 2.1. Animals

The animals used in this study included six species of odontocete cetaceans, in particular striped dolphin (*Stenella coeruleoalba*) (IHC 5/21), bottlenose dolphin (*Tursiops truncatus*) (IHC 1/2), common dolphin (*Delphinus delphis*) (IHC 1/1), harbour porpoise (*Phocoena phocoena*) (IHC 1/10), long-finned pilot whale (*Globicephala melas*) (IHC 1/1), and Cuvier’s beaked whale (*Ziphius cavirostris*) (IHC 1/2). Animals were found stranded along the Italian and Spanish and North Sea coastline and a full post-mortem examination following an internationally standardised procedure [17] was performed by trained personnel aiming at determining the cause of death and health status. A detailed list of the examined animals is reported in Appendix A Table A1.

### 2.2. Tissue Sampling and Processing

The details of tissue sampling and processing can be found in previous work (De Vreese et al., 2020 [16]), although not all samples contained the entire ear canal from the ear opening to the middle ear. Briefly, after sampling, specimens of the external ear canal and surrounding tissues were fixed in 10% neutral-buffered formalin and the time from fixation to tissue processing ranged from several days to 18 months. The far-most medial end of the ear canal, together with the TP-complex, was separated and decalcified using a commercial decalcifier (Biodec R, Bio-Optica^®^, Milano, Italy). Next, slabs with a diameter of about 3–4 mm were dissected, transverse to the local orientation of the ear canal longitudinal axis, embedded in paraffin, sectioned to a thickness of 4 µm, and mounted on polarized glass slides. Sections for staining with haematoxylin and eosin were obtained from all slabs and dried overnight at 70 °C, followed by automated staining using a Leica Autostainer XL (Leica Biosystems Nussloch GmbH, Milano, Italy). Few sections were stained with Masson’s trichrome with Aniline blue, or Alcian blue according to the standard laboratory protocols. Slides were coverslipped using a mixture of Eukitt^®^ (ORSAtec GmbH, Bobingen, Germany) and xylene.

### 2.3. Immunohistochemistry (IHC)

IHC staining was performed using an automatic immunostainer (Ventana Benchmark XT, Roche-Diagnostic, Mannheim, Germany), which uses a kit with a secondary antibody, and with a horseradish peroxidase (HRP)-conjugated polymer that binds the mouse and rabbit primary antibodies (ultraViews Universal DAB, Ventana Medical System, Mannheim, Germany). All reagents were dispensed automatically except for the primary antibody, which was dispensed by hand.

The following primary antibodies were used: monoclonal mouse anti-human CD3 (Dako M7254), monoclonal rabbit anti-human CD20 (Thermo Scientific #RB-9013-P, Runcorn, UK), polyclonal rabbit anti-human Iba-1 (Wako #019-19741), monoclonal mouse anti-human HLA-Dr (Dako M0746, Glostrup, Denmark), monoclonal mouse anti-porcine Vimentin (clone V9, Dako M0725, Glostrup, Denmark), monoclonal mouse anti-human Cytokeratin (Dako M3515, Glostrup, Denmark), and monoclonal rabbit anti-human von Willebrand factor (factor VIII) (Dako A0082, Glostrup, Denmark) (for details, see Table 1).

After staining, all slides were washed with standard dishwashing soap, rinsed with tap water several times, dehydrated in increasing concentrations of alcohol, coverslipped, and dried overnight at room temperature. The specificity of the immunohistochemical reaction was checked using white control sections (primary antibody absent) and positive controls selected for each antibody (for details see Table 1). All slides were examined with either an Olympus BX41 microscope (Olympus Italia S.r.l., Milan, Italy) or a Nikon Eclipse Ci-L (Nikon Instruments, Tokyo, Japan) at up to ×600 magnification, and scanned with a D-sight scanning microscope at ×400 magnification (A. Menarini Diagnostics, S.r.l., Florence, Italy). Digital images were uploaded to a server (Telepathology, Visia Imaging S.r.l., San Giovanni Valdarno, AR, Italy) and pictures were taken as screenshots from the online platform. Images were edited using Fiji software (ImageJ 1.52i) to add the scale bar, to adjust the brightness and contrast when appropriate, and montages were made using the Magic Montage plugin.

## 3. Results

**Figure 1:**
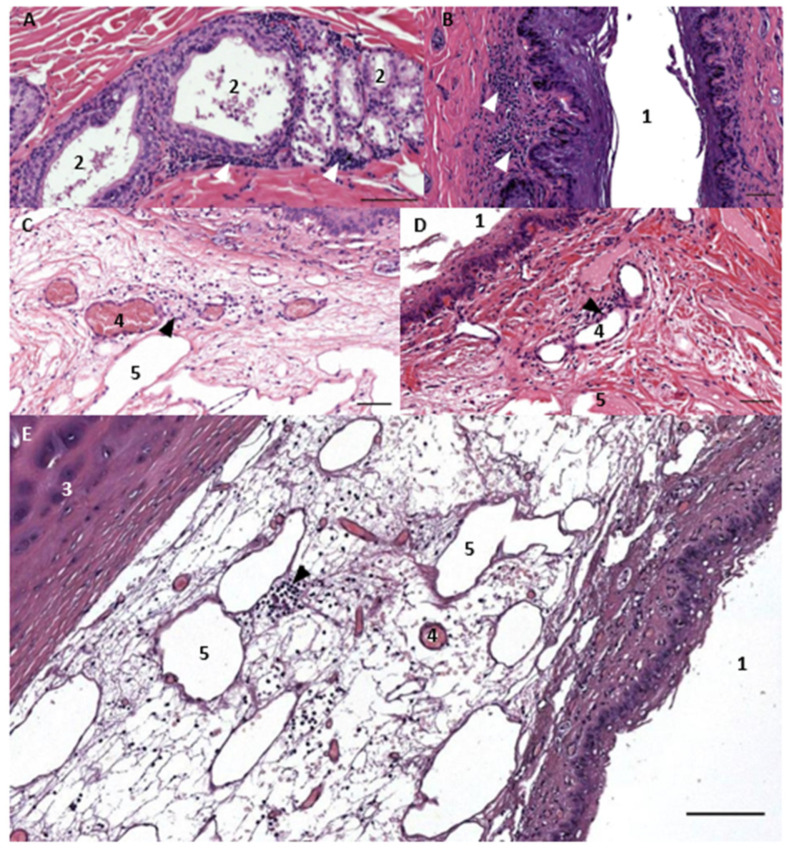
The histological cross-sections (HE staining) of the ear canal in a common dolphin (**A**) 169/17, and bottlenose dolphin (**B**) 457, at about 1 and 3 cm beneath the skin, respectively, and at about 4 cm beneath the skin in striped dolphin (**C**,**D**) 292/18 and (**E**) 274/18, at the level of the cartilage (3). Note the presence of glands and glandular ducts (2) and mononuclear cells (arrowhead). Scale bar (**A**) 100 µm, (**B**) 50 µm. Histological image (HE staining) of the mononuclear cells (lymphocytes) in the subepithelial tissue associated with the ear canal at the level of the glands (**A**–**C**), and in the reticular connective tissue network with blood vessels (4) and vascular lacunae (5) between the ear canal and cartilage. Scale bar top: (**A**,**C**,**D**) 100 µm; (**B**,**E**) 50 µm.

**Figure 2:**
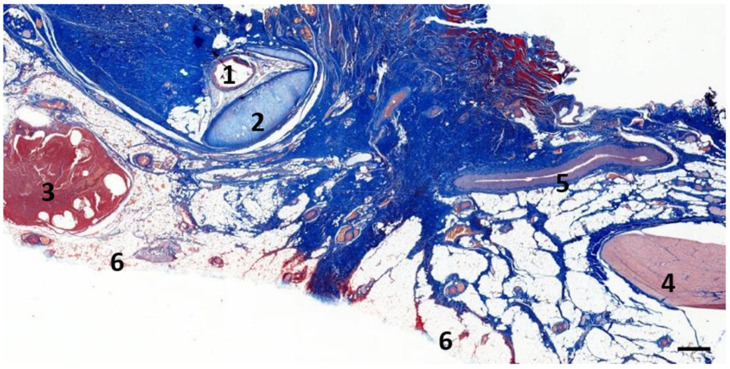
The histological image (Masson’s trichrome stain) of the left lateral view of a parasagittal section through the region of the left external ear canal of a striped dolphin (ID127565) at about 4 cm beneath the skin. 1: External ear canal; 2: Cartilage of the ear canal; 3: Lymph node; 4: Facial nerve; 5: Facial vein; 6: Adipose tissue. Scale bar = 1 mm.

Two types of lymphoid tissue associated with the ear canal were characterized. The first were loosely arranged mononuclear cells in the cetacean ear canal’s subepithelial tissue in two locations, one at the level of the glands, and the other close to the middle ear in between the ear canal lumen and the cartilage (Figure 1). In both locations, there was a multifocal presence of lymphocytes, with scant plasma cells and macrophages. This diffuse lymphoid tissue was considered to form part of the MALT (mucosa-associated lymphoid tissue) and was labelled as ECALT (ear-canal associated lymphoid tissue). The second type of lymphoid tissue was a clearly defined, relatively large, nodular lymphoid tissue, likely part of the mandibular lymph node group, situated ventrocaudal to the ear canal at the level where the facial nerve crosses the ear canal ventrally (Figure 2).

### 3.1. Ear Canal-Associated Lymphoid Tissue (ECALT)

**Figure 3:**
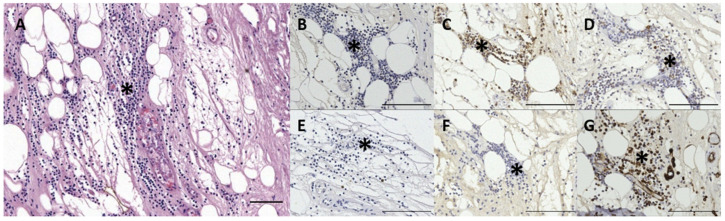
The histological images: (**A**) HE staining; (**B**) CD3; (**C**) CD20; (**D**) HLA-DR; (**E**) Iba-1; (**F**) PanCK; (**G**) Vimentin of a mononuclear infiltrate (asterisks) between the left ear canal and cartilage in a bottlenose dolphin (444_L18). Scale bars = 50 µm.

**Figure 4:**
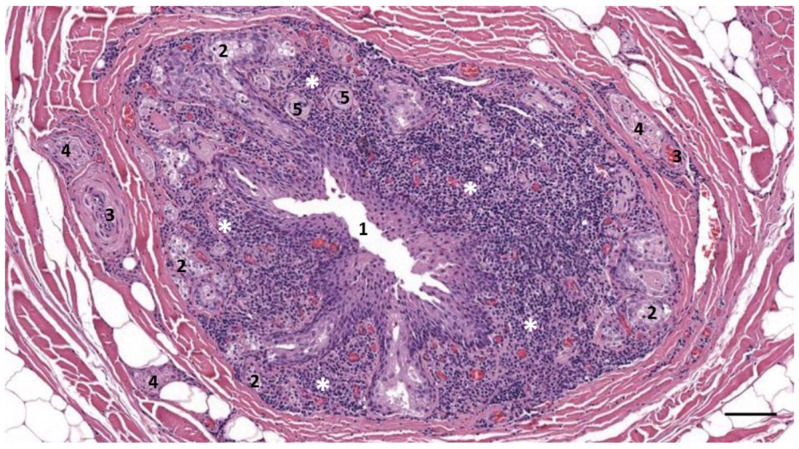
The histological cross-section (HE staining) of the left ear canal in a striped dolphin (293/18_L3). There as inflammation of the ear canal (1), with the abundant presence of mononuclear cells (asterisk) in the subepithelial tissue and among glandular structures (2). 3: Vasculature; 4: Nerve; 5: Lamellar corpuscle. Scale bar = 100 µm.

**Figure 5:**
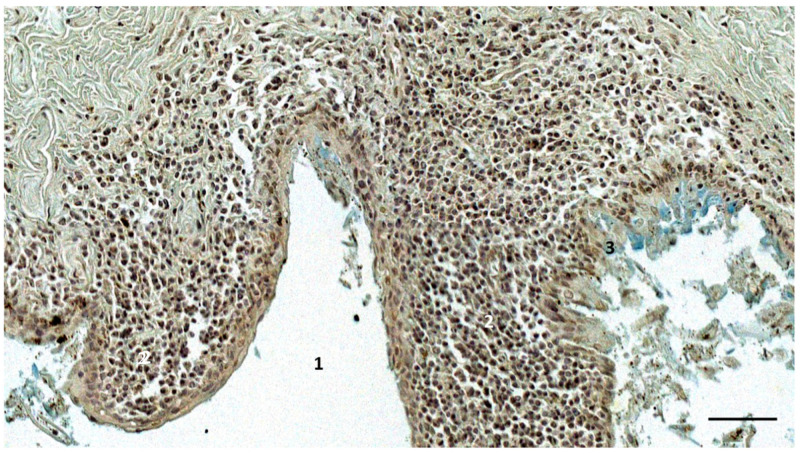
The histological cross-section (Alcian blue staining) of the right ear canal (1) of a bottlenose dolphin, at about 2 cm beneath the skin (ID444_R5). Note the subepithelial abundant presence of mononuclear cells (2), and the abrasive epithelium of the ear canal (3), showing a blue staining. Scale bar = 50 µm.

**Figure 6:**
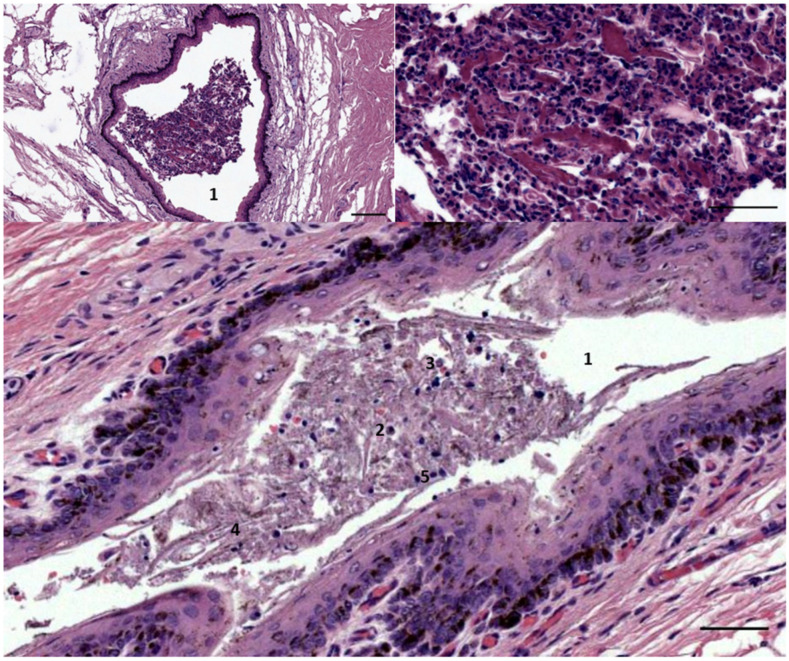
The histological images (HE staining) of a transverse section through the ear canal (1) of two striped dolphin (top ID2926 bottom 488/17). The images show the presence of inflammatory cells such as macrophages (2), neutrophils (3), desquamated epithelial cells (4), and cells with pyknotic nuclei (possible glandular cells) (5), within the lumen of the canal. Scale bars: top left = 100 µm, top right = 50 µm, bottom = 50 µm.

The ECALT was present in all specimens in two locations: superficial and deep. It was noted at the level of the glands, which in small delphinids is about 1 to 1.5 cm from the external ear opening, and deeper into the tissue between the ear canal lumen and the ear canal cartilage. In both locations and all species, there was a resident population of dispersed macrophages as identified by the anti-Iba-1, rare plasma cells, and lymphocytes. In most specimens, the localisation of lymphocytes was sparse, part of a resident population, and part of a first barrier immune system, similar to the resident mononuclear cells in the dermal papillae around the external ear opening. In other cases (ID444, 293/18, and 488/17), there was an evident activation of the tissue, forming accumulations of mononuclear cells in the papillary layer and with signs of inflammation (Figure 3, Figure 4, Figure 5 and Figure 6).

The lymphocytic population showed variations in immunoreactivity (IR) to the anti-CD20 and anti-CD3 antibodies. In non-activated ECALT tissue, two (ID77/18, 169/17) out of eight cases (five superficial tissue, three deep tissue) showed IR to anti-CD20, while none of the specimens showed IR to anti-CD3. In five cases of reactive lymphoid tissue (four superficial, one deep), all showed IR to CD20, with a variation in the IR, while one bottlenose dolphin showed IR to CD3 in a minor part of the lymphoid cell population. It is not known how these T- vs. B-cell differences are explained, although they could depend on the type of infective agent and the chronicity of the inflammatory infiltrate. In three of four cases with reactive tissue, the identified lymphoid cells also presented IR to anti-HLA-DR (dispersed macrophages, similar to non-reactive tissue) and anti-vimentin (Figure 3). One exception was the inflammation in the long-finned pilot whale, in which the lymphocytes did not show IR to anti-vimentin.

The ECALT contained a resident population of immune cells consisting of mature B-lymphocytes concentrated in patches in the two distinct locations of the ear canal and macrophages dispersed in the subepithelial tissue around the ear canal.

### 3.2. Nodular Lymphoid Tissue

**Figure 7:**
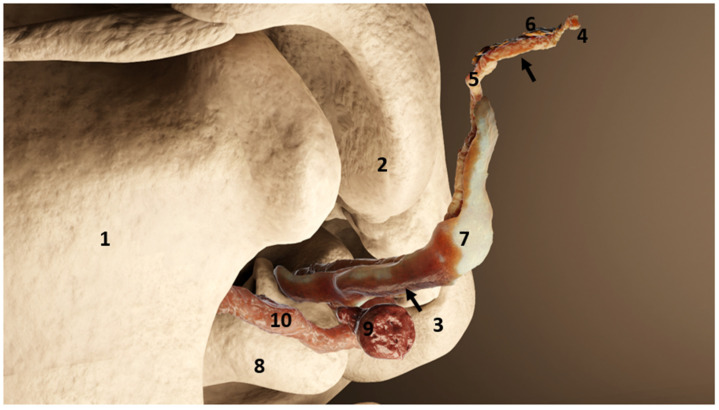
The 3D rendering demonstrating the nodular lymphoid tissue associated with the left ear canal in a generic dolphin (rostrolateral view). The nodular tissue is situated ventr(orostr)al to the ear canal and closely associated with the facial nerve (8). 1: Mandible; 2: Retroarticular process of the squamosal; 3: Exoccipital; 4: Ear canal opening at the level of the skin; 5: Ear canal epithelium; 6: Ear canal glands; 7: Ear canal cartilage; 8: Tympanic bone; 9: Nodular lymphoid tissue; 10: Facial nerve; The arrows indicate the two locations of samples used for immunohistochemical analysis: superficial at the level of the glands, and deep at the level of the cartilage and the lymph node.

**Figure 8:**
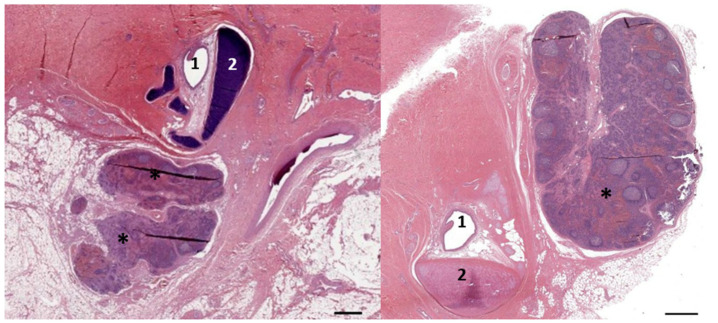
A histological image (HE stain) (274/18_R9). Overview of the nodular lymphoid tissue (asterisks) in the close vicinity of the left and right external ear canal (1) and cartilage (2). Scale bars = 1 mm.

**Figure 9:**
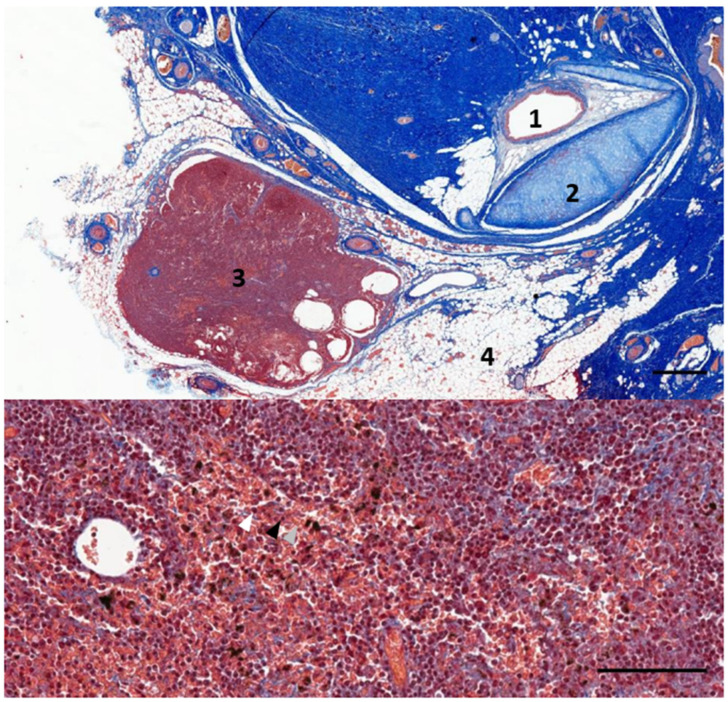
A histological image of Masson’s trichrome staining of a parasagittal section through the region of the left external ear canal of a striped dolphin (ID127565). The bottom image shows the detail of the nodular tissue showing the presence of red blood cells (grey arrowhead), lymphocytes (black arrowhead), and macrophages (white arrowhead). 1: External ear canal; 2: Cartilage of the ear canal; 3: Lymph node; 4: Fat tissue. Scale bar top = 1 mm, bottom = 100 µm.

**Figure 10:**
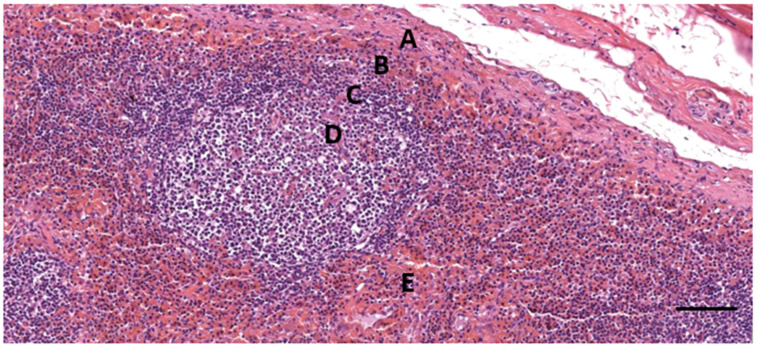
A histological image (HE stain) (274/18_R9). Detail of the cortical zone with a thin connective tissue capsule (A), a subcapsular sinus (B) with many erythrocytes, a follicle with mantle zone (C) and germinal centre (D), and parafollicular tissue (E) with a dense concentration of erythrocytes. Scale bar = 100 µm.

**Figure 11:**
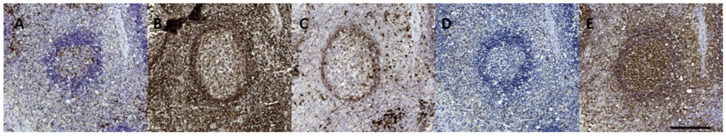
A histological image (274/18_8R) of the immunohistochemical analysis of a lymphoid follicle stained with (**A**) anti-CD3, (**B**) anti-CD20, (**C**) anti-HLA-DR, (**D**) anti-Iba1, (**E**) anti-vimentin (**E**). Scale = 100 µm.

In five samples of the external ear canal from four striped dolphins, nodular lymphoid tissue was seen to be closely associated with the external ear canal. All lymph nodes were situated rostroventral to the ear canal, in the dorsal margin of the mandibular fat body, about 3–4 cm beneath the skin, bilaterally, a few millimetres lateral to the passing facial nerve (Figure 2 and Figure 7). The lymph node morphology was similar to the general mammalian somatic structure, although with certain peculiarities. The lymphoid tissue was multinodular, with sizes ranging from 1 to 10 mm in diameter (Figure 8). They were partly surrounded by a thin connective tissue capsule with trabeculae radiating into the lymph node parenchyma dividing the node into lobes, although the cortex and medulla were not always easily distinguishable, and with variation in the organisation into follicles and parafollicular tissue. The capsule was thin in some areas to absent in other areas of the nodes, showing a direct transition to the surrounding fat and connective tissue of the acoustic fat body (Figure 8 and Figure 9). Where present, the capsule consisted of dense connective collagenous tissue with vasculature, innervation, and little adipose tissue, and the occasional presence of mechanosensitive lamellar corpuscles. No smooth muscle tissue was noted. Where there was a capsule, there was also a subcapsular sinus with red blood cells and little loose connective tissue (Figure 10). The parenchyma consisted of the cortex with lymphoid follicles with mantle zone and germinal centre and parafollicular tissue/medulla. The follicle’s centre showed IR to both anti-CD3 and anti-CD20, indicating the presence of both T- and B-cells. The mantle zone was more cell-dense, and IR to anti-CD20 and anti-HLA-DR, indicating the presence of both B-cell and antigen-presenting cells such as macrophages (Figure 11B,C). Next, there was a paracortical/marginal zone with anti-CD3 IR, although with differences between follicles (Figure 11A, central follicle vs. top left of image part of different follicle with more IR in paracortical zone). The lymph node parafollicular tissue/medulla consisted of patched to diffuse lymphoid tissue and interconnected areas of medullary chords, although not always clear (Figure 11). The cellular composition consisted of B- and T-lymphocytes, plasma cells, reticular cells (with reticular connective tissue between cells), macrophages, neutrophils, and eosinophils, with IR to all antibodies used, respectively.

**Figure 12:**
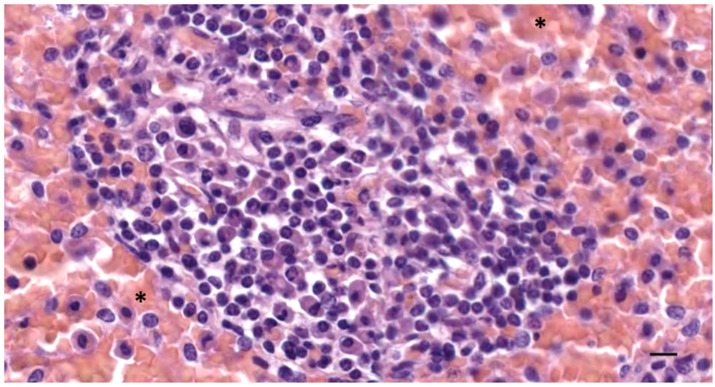
A histological image (HE stain) (274/18_R9). Patched follicle tissue and the intense presence of red blood cells (asterisks). Scale bar = 10 µm.

**Figure 13:**
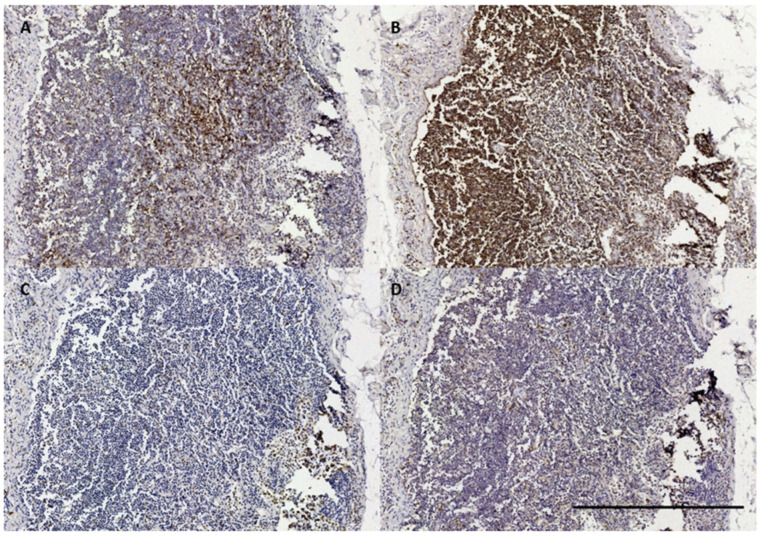
The histological images of IHC in a striped dolphin nodular lymphoid tissue (ID362/18). (**A**) CD3; (**B**) CD20; (**C**) Iba-1; (**D**) vWf. Scale bar = 500 µm.

Aside from these cells, there was also a large number of erythrocytes dispersed in the entire organ although they were concentrated in specific areas such as the subcapsular sinuses and parafollicular tissue (Figure 9, Figure 10 and Figure 12). In the stroma, between the main lobes of lymphoid tissue, there were arteries and veins, and also a few detectable lymph vessels in the parenchyma. No signs of high-endothelial venules (HEV) were present as analysed with anti-vWf (Figure 13).

### 3.3. Immunoreactive Tissue

Several animals presented pathological alterations in and around the ear canal, either directly associated with the ear canal such as otitis externa or as indirect findings associated with a general/systemic pathology. The results are summarised in Table 2.

In the striped dolphin specimens (#21), more than a third presented some kind of pathological changes in or around the external ear canal, while three individuals presented an inflammation of the external ear canal (otitis externa) (Figure 6). Other findings included a circummeatal haemorrhage, panniculitis, and or dermatitis, muscle-related pathologies, and indications of a cholesteatoma, epithelial cyst, and/or keratoma.

**Figure 14:**
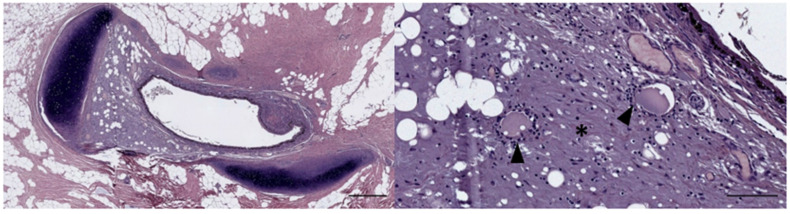
A histological image (HE staining) of a transverse section through the external ear canal of a harbour porpoise, about 4 cm beneath the skin (UT1718_L1402). Note the perivascular presence (cuffing) of lymphocytic cells and the presence of macrophages (arrows); oedema (asterisk) with protein leakage associated with an inflammatory process, with splitting of collagen fibres. Scale bar left = 500 µm, right = 100 µm.

None of the 10 harbour porpoise individuals from the North Sea that were subjected to histological evaluation presented alterations directly associated with the external ear canal. The only abnormality involved a single specimen with unilateral congestion with contracted arteries, focal acute muscle degeneration, perivascular presence (cuffing) of the lymphocytes and macrophages, and oedema between the ear canal and the cartilage in the deepest parts of the ear canal (Figure 14).

Otitis externa was found in three striped dolphins, a bottlenose dolphin, one of two Cuvier’s beaked whales and a long-finned pilot whale specimen. Two animals presented bilateral inflammation, one unilateral, and for the others, it was not known since only one of the two ear canals was studied histologically. It was not always clear whether there was inflammation, a simple activation of the diffuse ECALT, or the presence of the non-activated resident ECALT but in higher proportions than in the other animals.

**Figure A1:**
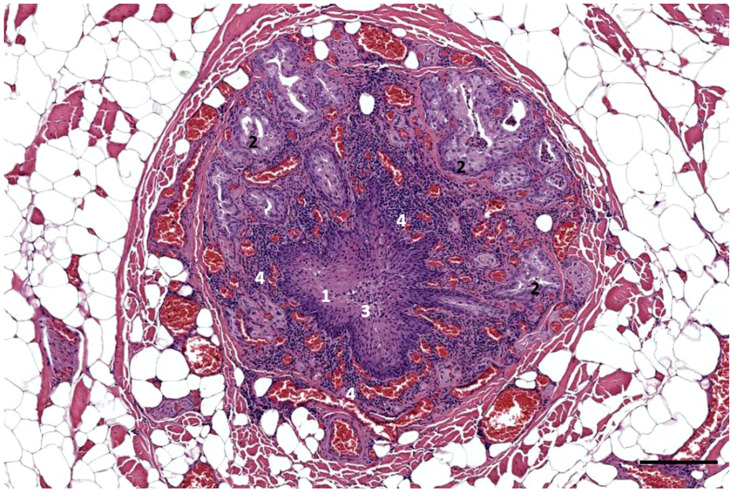
A histological image (HE staining) of a transverse section through the external ear canal (1) of a striped dolphin (293/18) at the level of the glands (2). Note the inflammation with a compressed ear canal lumen that is filled with inflammatory cells and sloughed epithelial cells (3), the circummeatal presence of mononuclear cells (4) and the overall congestion of the tissue. Scale bar = 200 µm.

**Figure A2:**
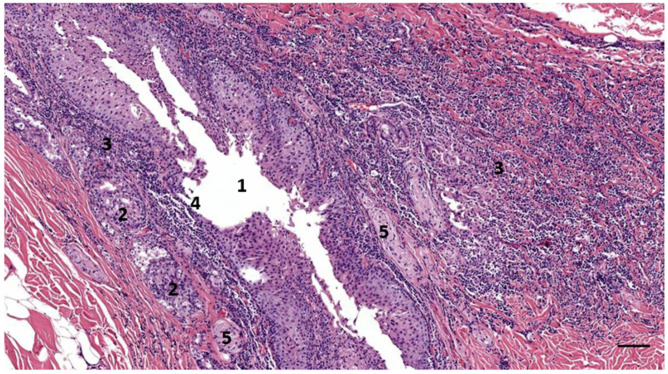
A histological image (HE staining) of an oblique section through the external ear canal (1) of a striped dolphin (293/18) at the level of the ventral curvature of the canal and glands (2). Note the intense circummeatal presence of inflammatory cells (3) with ulceration of the epithelium (4), and the presence of lamellar corpuscles (5). Scale bar = 100 µm.

**Figure A3:**
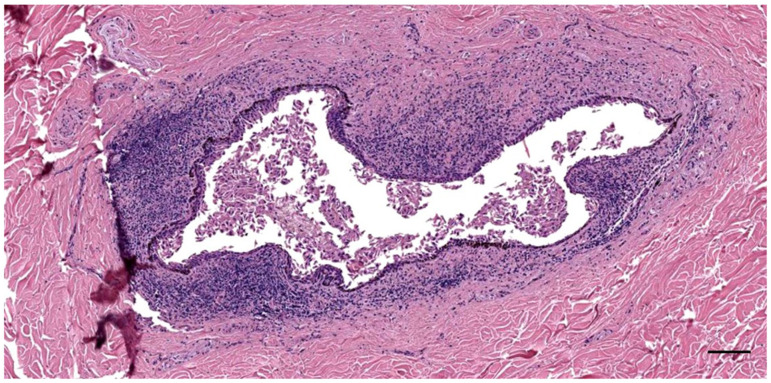
A histological image (HE staining) of a transverse section through the external ear canal of a long-finned pilot whale (441) at about 5 cm beneath the skin. The ear canal contains inflammatory cells and epithelial cells, while there is the circummeatal presence of mixed inflammatory cells. Scale bar = 100 µm.

All inflammatory findings were chronic, characterized by the presence of a multifocal mixed cell inflammatory reaction in the subepithelial tissue, often in the superficial half of the canal from the skin down to the medial end of the ventral curvature where the canal turns horizontal again. In three striped dolphins and the long-finned pilot whale, the otitis externa was purulent with the presence of viable and hypersegmented neutrophils, macrophages, and lymphocytes in both the submucosal tissue and the lumen of the ear canal (Figure 6) (See also Appendix B, Figure A1, Figure A2 and Figure A3). Concurrent findings included (a) epithelial changes with hyperplasia, apoptotic changes, and ulceration with leakage of melanin the subepithelial tissue; (b) adenitis; and (c) dermatitis and/or panniculitis.

## 4. Discussions

### 4.1. Ear Canal-Associated Lymphoid Tissue

The lymphoid tissue associated with the ear canal was presented in three locations and in various states of activation. The first is the circummeatal presence of mononuclear cells at the level of the glands, in the subepithelial layers, scarce and in low concentrations, although it is sometimes visible in clusters. It likely considers a resident population forming part of a first barrier immune system, and which could also be noted in the activated state in some animals (the case of the long-finned pilot whale). The second is the presence of mononuclear cells in the medial half of the canal between the ear canal and the cartilage, also as a resident population or activated state. The third would be the more or less well-defined lymphoid tissue situated ventral to the ear canal before it reaches the tympanic bone, at about the level of the facial nerve crossing.

The presence of lymphoid cells in the lamina propria of the ear canal of odontocete cetaceans has been reported in a few cases as a full circummeatal lymphoid organ (Baird’s beaked whale (*Berardius bairdii*), sperm whale (*Physeter macrocephalus*), and pygmy sperm whale (*Kogia breviceps*)) [24], which was described in Baird’s beaked whale as subepithelial lymph follicles with “many crypts and lymphoid infiltrations of the epithelium, which has transformed into a peculiar lymphoid organ similar to the tonsils”. According to this description, it is the epithelium itself that shows transformation, and which, together with a subepithelial infiltration of lymphocytes, forms a well-defined lymphoid organ. It is not entirely clear as to what extent this interpretation should be taken, whether the epithelium itself was involved, and whether the subepithelial lymphoid infiltrations formed the tonsil-like organ. In this study, there was never any intraepithelial infiltration with lymphoid cells, although the subepithelial infiltration was a common finding. In the sperm whale, the extensive peri-meatal subepithelial lymphoid organ at the level of the blubber layer was divided into lobules with deep crypts and many germinal centres [24]. In our study, a combination of those descriptions was found, although with differences such as the subepithelial tissue forming follicles, but never as well-defined as a tonsil-like organ, which would be the actual lymph node. In any case, the findings by Yamada [24] likely refer to the activated state of the circummeatal lymphoid tissue, which is consistent with what we found in this study: subepithelial lymphoid tissue was present in almost all specimens, although activated in some and latent in others. We encourage further multi-species studies to investigate any intraspecific differences.

The lymphoid tissue in the subepithelial layer forms part of the mucosa-associated lymphatic tissue (MALT), which comprises, among others, the gut-, nasal-, bronchus-associated lymphatic tissue. It was not organized as a well-defined structure or mass, but rather diffuse, making it part of the diffuse mucosa-associated lymphatic tissue (D-MALT). Lymphocytes associated with glandular structures occur in various cetacean mucosae such as the nasal sac system [25], the larynx [26], intestines [6], anus [27], and in the skin [11]. The lymphocytic populations in the connective tissue between the ear canal and cartilage, although here also considered to form part of the same ear canal-associated lymphoid tissue, were not spatially associated with glandular structures, but rather with vascular lacunae, indicative of an intricate relation with systemic functions.

Similar to the MALT, the cellular composition of the ECALT consisted of T-cells and B-cells as well as plasma cells and macrophages. The variation in cellular IR to anti-CD20 and other antibodies could be explained by cellular autolysis and the fixation of the tissue, associated with the time of fixation after death as well as the tissue type (e.g., the superficial tissue showed better fixation than the tissue at the level of the cartilage in all samples). The MALT in the skin of a foetus and juvenile *Sotalia guianensis* and a juvenile *G. macrorhynchus* appeared in clusters of T-cells ‘surrounded by B-cells’, showing a similar image to the single case of a bottlenose dolphin with active ECALT in our studies [11].

The pathological findings described here were generally considered to be associated with a local cause such as otitis externa, while others were consistent general pathological findings not included in this paper.

### 4.2. Ear Canal Lymph Node

The lymphonodal tissue described here was activated in all specimens, although its identification was not conclusive as to whether it belonged to a predescribed lymphocentre. Enlarged tissue is easier to include in the opportunistic samples of the external ear canal. Given the small size of the nodules, it might be complicated to macroscopically detect the lymph node under non-pathological conditions, but more investigations should be conducted to include the tissue of the fat body ventral to the ear canal in which the nodular tissue was found, to see whether these nodes are present in all species and in non-activated physiological states. There have been several references to lymph nodes in the area, ranging from nodes relatively caudal to the external ear canal, around the brachiocephalic muscle, and called the dorsal superficial cervical lymph nodes [6,28], mandibular lymph nodes [7] and parotid lymph node (probably only from topography since the gland is absent in the family [7,29]. The location of the lymph node was topographically consistent with the parotid lymph node found in terrestrial mammals. However, if such a homology were to be made, a different name could be sought in this case. According to Baum [30], the parotid, mandibular, and retropharyngeal centres are the three lymph centres of the head. The parotid lymph node is located just caudal to the temporomandibular joint, partially covered by the parotid gland, with the anterior portion lying on the caudal margin of the mandible, thinly covered by subcutaneous fat. It is located at the crossing of the dorsal buccal nerve and the zygomatic branch of the facial nerve. This description is consistent with our findings. The mandibular centre is more closely related to more cranial areas but does reach areas of the temporomandibular joint and parotid area, while the inconstant lateral retropharyngeal lymph node is situated more caudally but shows a closer spatial relation to the ear canal [30].

The morphology of the Tissue corresponded largely to previous descriptions of mammalian somatic lymph nodes and other cetacean lymph nodes [7], except for the presence of a large amount of red blood cells throughout the node. Cetacean lymph nodes have been described as having a connective tissue capsule, with or without the presence of smooth muscle fibres [1,6,9,31]. Visceral lymph nodes seem to present smooth muscle, while somatic lymph nodes do not [1]. The thickness of the capsule can vary between locations and species [7,31]. To our knowledge, this study provides the first description of a partial absence of the capsule.

In comparison, lymph nodes in the harbour porpoise and common dolphin and the mesenteric lymph node in the striped dolphin have been described as having an inverse structure with the follicles situated central in the node, surrounded by medullar peripheral sinusoids and cords [29,31,32]. Other differences included no clear distinction between the cortex and medulla, and the heterogeneity in the general structure such as with the distribution of the erythrocytes, patchy zones in the parafollicular tissue, and the partial absence of a connective tissue capsule made the organ resemble the cetacean spleen or haemolymph node more than a traditional lymph node. The IR to anti-vWf did not show the presence of high-endothelial venules as described in the haemolymph nodes, although this is not an exclusive factor. While there are no descriptions of the presence of haemolymph nodes in cetaceans, this could be an explanation of the findings, as they have not only been described in ruminants, but have also been described in rats and humans [33]. As such, cetacean lymph nodes, aside from their role in the immune system, could also be charged with the breakdown of old red blood cells [34]. Among the various explanations for the presence of red blood cells inside the lymph node, we also considered haemorrhage, possibly associated with a traumatic event, sinus erythrocytosis associated with a haemorrhage in nearby tissue, and dissection-induced artefacts. However, interpretation should be taken with caution because of the post-mortem degeneration of the soft tissues.

## 5. Conclusions

This manuscript presents results on the morphological and immunophenotypical organisation of the ECALT and ear canal-associated lymph nodes. It contributes to the identification and mapping of the occurrence of diffuse and nodular lymphoid tissue in the body of odontocete cetaceans and to the knowledge on its cellular composition. The ECALT consisted of two patches of lymphoid tissue, one associated with the auricular glands, and one associated with the cartilage. The resident cell population was identified as B-lymphocytes. Additionally, the presence of an ECALT among all studied species and specimens supports the findings that the external ear canal lumen presents a continuation with the external environment.

Six out of 36 animals presented otitis externa with lymphocytic proliferation and in one case with the concurrent presence of T-lymphocytes, although the interpretation should be taken with caution. We also mention other pathologies related to the ear canal, and the presence of active lymph nodes situated ventral to the ear canal. The lymph node structure was largely similar to other mammalian somatic lymph nodes, although with specific characteristics and a distinct large amount of intramodular red blood cells of unknown aetiology. The number of individual animals that presented pathological alterations associated with the ear canal stresses the relevance of the ECALT and nodes in the odontocete immune system. In this regard, there were seemingly interspecific differences, although the number of samples did not permit a quantitative study related to the general pathology, species, or geography. Further investigation is needed to assess these differences, and the authors promote the sampling of the external ear canal and associated tissues in the general post-mortem analysis of wildly stranded animals.

## Figures and Tables

**Table 1:** The primary antibodies.

Antibody	Manufacturer/Code	Target	Cellular Staining	References	Mono/Polyclonal + Host	Antigen Origin	Dilution	Blocking	Positive Control
anti-CD3 *	Dako M7254	(Mature) T-lymphocytes	Cytoplasm and/or cell membrane	[5,18]	Monoclonal Mouse	Human	1:50	No	Canine + bottlenose dolphin lymph node
anti-CD20 *	Thermo Scientific #RB-9013-P	Mature B-lymphocytes	Cytoplasm and cell membrane	[4,5,18]	Monoclonal Rabbit	Human	1:800	No	
anti-Iba-1 *	Wako #019-19741	Macrophages and microglia	Cell membrane	[5,18]	Polyclonal Rabbit	Human	1:80	No	
anti-HLA-DR *	Dako M0746	B-cells, activated T-cells, macrophages, APC *	Cell-surface membrane (also cytoplasm)	[4,5]	Monoclonal mouse	Human	1:50	Yes	
**anti-Vimentin**	Dako M0725	Mesenchymal cells	Cytoplasm	[19,20,21]	Monoclonal mouse	Porcine	1:150	No	Internal: interstitial cells
anti-Cytokeratin (PanCK *)	Dako M3515	Epithelium	Cytoplasm	[20]	Monoclonal Mouse	Human	1:100	No	Internal: epithelium of external ear canal
anti-vWf *	Dako A0082	Endothelial cells	Cytoplasm	[22,23]	Monoclonal Rabbit	Human	1:300	No	Internal: blood vessel endothelium

* APC—Antigen-presenting cells including dendritic cells, B-lymphocytes, monocytes, macrophages, Langerhans cells. CD—cluster of differentiation; HLA—human leukocyte antigen, a.k.a. major histocompatibility complex (MHA); Iba-1—Ionised calcium-binding adaptor molecule-1; PanCK—pan-cytokeratin; vWf—von Willeband factor.

**Table 2:** An overview of the pathology findings and the number of cases.

Pathology	Species	Positive	Total Animals	Percentage
Otitis externa	Tt	1	2	50%
	Sc	3	21	15%
	Gm	1	1	100%
	Zc	1	2	50%
	Pp	0	10	0.00%
	**TOTAL**	**6**	**36**	**16.67%**
Haemorrhage	Sc	3	21	14.29%
Erythrocytes in external ear opening	Sc	2	21	9.52%
	Pp	1	10	10.00%
Panniculitis/dermatitis	Sc	3	21	14.29%
	Gm	1	1	100.00%
Vasculitis	Sc	1	21	4.76%
Muscle atrophy/degeneration	Sc	8	21	38.10%
	Tt	1	2	50.00%
	Pp	1	10	10.00%
Peripheral nerve pathology	Sc	3	21	14.29%
Epithelial cyst/Cholesteatoma/Keratoma	Sc	2	21	9.52%

## Data Availability

Not applicable.

## Appendix A

**Table A1:** The animals used in this study. CC—conservation code (according to IJsseldijk et al., 2019 [17]); HC: histochemistry; IHC: immunohistochemistry; Y: Yes; N: No; M: Male; F: Female; na: not available.

Animal ID	Species	CC	Origin	Date of Necropsy	Death	Sex	Length (CM)	Weight (KG)	Ear Canal		Lymph Node	Lab Analysis Lymphoid Tissue
									Left	Right		
**N-00419-16**	*S. coeruleoalba*	2	Port de la Selva, Spain	16 November 2016	Spont.	M	103	14	Y	N	N	HC
**N-00044-17**	*S. coeruleoalba*	2	Port Ginesta, Spain	10 February 2017	Spont.	M	153	37	Y	Y	N	HC
**N-00168-17**	*S. coeruleoalba*	2	L’Escala, Spain	19 April 2017	Spont.	M	193	79	Y	N	N	HC
**N-00169-17**	*D. delphis*	2	L’Escala, Spain	20 April 2017	Spont.	M	179	72	Y	Y	N	HC, IHC
**N-00488-17**	*S. coeruleoalba*	2	Gavà, Spain	28 September 2017	Euth.	F	198	70	Y	Y	N	HC
**N-00509-17**	*S. coeruleoalba*	2-3	Tarragona, Spain	10 October 2017	Spont.	M	183	87	Y	Y	N	HC
**12691**	*S. coeruleoalba*	2	Bibona, Livorno, Italy	19 December 2017	Spont.	M	193,5	62.4	Y	Y	N	HC
**12693**	*S. coeruleoalba*	3	Livorno Italy	19 December 2017	Spont.	F	160	40	Y	Y	N	HC
**12694/429**	*Z. cavirostris*	2	Livorno Italy	23 December 2017	Spont.	M	503	na	Y	Y	N	HC, IHC
**12703/2926**	*S. coeruleoalba*	2	Bagni Buraxen, Imperia, Italy	13 January 2018	Spont.	M	133	34.5	Y	Y	N	HC
**N-00021-18**	*S. coeruleoalba*	2-3	Calella de palafrugell, Spain	15 January 2018	Spont.	M	182	73	Y	N	N	HC
**12708/5386**	*S. coeruleoalba*	2	Genova Italy	21 January 2018	Spont.	M	192	69	Y	Y	N	HC
**N-00042-18**	*S. coeruleoalba*	2	Viladecans, Spain	25 January 2018	Spont.	M	178	62	Y	N	N	HC
**N-00077-18**	*S. coeruleoalba*	2	Delta del Ebro, Spain	19 February 2018	Spont.	M	220	97.5	Y	Y	N	HC, IHC
**441**	*G. melas*	4	Aglientu, Sardegna, Italy	20 March 2018	Spont.	M	525	na	Y	Y	N	HC, IHC
**444**	*T. truncatus*	3	Pellestrina, Venezia, Italy	24 March 2018	Spont.	M	264	260	Y	Y	N	HC, IHC
**N-00145-18**	*S. coeruleoalba*	2	Palamós, Spain	26 March 2018	Spont.	F	195	91	Y	N	N	HC
**N-00232-18**	*S. coeruleoalba*	1	Tarragona, Spain	24 May 2018	Spont.	M	180	51.5	Y	N	N	HC
**N-00274-18**	*S. coeruleoalba*	3	Delta del Ebro, Spain	24 June 2018	Spont.	M	152	38	Y	Y	Bilateral	HC, IHC
**N-00292-18**	*S. coeruleoalba*	2	Tarragona, Spain	6 July 2018	Spont.	M	194	59.5	Y	Y	N	HC
**N-00293-18**	*S. coeruleoalba*	3	Port Ginesta, Spain	09/07/2018	Euth.	F	187	85	Y	Y	N	HC, IHC
**N-00620-17**	*S. coeruleoalba*	2	Vilanova i la Geltrú, Spain	24 July 2018	Spont.	M	196	75.5	Y	N	N	HC
**N-00362-18**	*S. coeruleoalba*	2	Riumar, Spain	12 September 2018	Euth.	M	181	78	Y	Y	Left	HC, IHC
**109064/449**	*S. coeruleoalba*	2	Groticelle, Calabria, Italy	13 October 2018	Spont.	M	102	12.23	Y	Y	Right	HC, IHC
**N-00177-19**	*Z. cavirostris*	4	Tossa de Mar, Spain	20 April 2019	Spont.	M	520	1200	Y	Y	N	HC
**127565**	*S. coeruleoalba*	2	Calabria, Italy	28 November 2018	Spont.	M	200	na	Y	Y	N	HC
**AE565/457**	*T. truncatus*	2	Bibione, Venezia, Italy	29 January 2019	Spont.	M	285	260	Y	Y	N	HC, IHC
**UT1664**	*P. phocoena*	2	Ouddorp, the NLs	9 July 2018	Spont.	F	92	11	Y	N	N	HC
**UT1692**	*P. phocoena*	1	Kats, the NLs	25 July 2018	Spont.	M	90	12.5	Y	N	N	HC
**UT1709**	*P. phocoena*	2	Oostkapelle, the NLs	19 September 2018	Spont.	M	95	10.5	Y	Y	N	HC, IHC
**UT1711**	*P. phocoena*	2	Petten, the NLs	25 September 2018	Spont.	M	146	37.5	Y	Y	N	HC
**UT1712**	*P. phocoena*	3	Dishoek, the NLs	4 October 2018	Spont.	M	124.5	30.5	Y	Y	N	HC
**UT1718**	*P. phocoena*	2	Vlieland, the NLs	05 January 2019	Spont.	M	113	24	Y	N	N	HC
**UT1727**	*P. phocoena*	2	Ijmuiden Seport Marina, the NLs	13 January 2019	Spont.	F	141	37	Y	Y	N	HC
**UT1728**	*P. phocoena*	1	Egmond, the NLs	17 January 2019	Spont.	F	155	64.5	Y	Y	N	HC
**UT1734**	*P. phocoena*	1	Terschelling, the NLs	27 January 2019	Spont.	M	103	24.5	Y	Y	N	HC
**UT1740**	*P. phocoena*	1	Terschelling West, the NLs	27 February 2019	Spont.	M	102	22.2	Y	Y	N	HC
